# Changes in the Intestinal Microbiota of Patients with Inflammatory Bowel Disease with Clinical Remission during an 8-Week Infliximab Infusion Cycle

**DOI:** 10.3390/microorganisms8060874

**Published:** 2020-06-09

**Authors:** Gyeol Seong, Namil Kim, Je-Gun Joung, Eun Ran Kim, Dong Kyung Chang, Jongsik Chun, Sung Noh Hong, Young-Ho Kim

**Affiliations:** 1 Department of Medicine, Samsung Medical Center, Sungkyunkwan University School of Medicine, 81 Irwon-ro, Gangnam-gu, Seoul 135-710, Korea; gyeol.seong@gmail.com (G.S.); er.kim@samsung.com (E.R.K.); do.chang@samsung.com (D.K.C.); 2 ChunLab, Inc., Nambusunhwan-ro, Seocho-gu, Seoul 06725, Korea; namil.kim@chunlab.com (N.K.); jchun@chunlab.com (J.C.); 3 Samsung Genome Institute, Samsung Medical Center, 81 Irwon-ro, Gangnam-gu, Seoul 06351, Korea; jegun.joung@samsung.com

**Keywords:** inflammatory bowel disease, intestinal microbiota, infliximab, mucosal healing

## Abstract

This study investigated changes in the intestinal microbiota during 8-week infliximab maintenance therapy in inflammatory bowel disease (IBD) patients in clinical remission. Microbial compositional differences were analyzed according to the trough level of infliximab (TLI) and mucosal healing (MH) status. 16S rRNA gene-based microbiome profiling was performed on 10 and 74 fecal samples from 10 healthy volunteers and 40 adult IBD patients, respectively. Fecal sampling occurred at 1–2 weeks (1W) and 7–8 weeks (7W) after infliximab infusion. TLI was measured by ELISA at 8 weeks, immediately before the subsequent infusion; MH was evaluated by endoscopy within 3 months. There were no significant changes in microbial composition, species richness, or diversity indices between 1W and 7W. However, 7W samples from the patients with TLI ≥ 5 μg/mL showed an increased species richness compared with patients with TLI < 5 μg/mL, and patients with MH showed increased diversity compared with non-MH patients. Beta-diversity analysis showed clustering between samples in the MH and non-MH groups. LEfSe analysis identified differential composition of *Faecalibacterium prausnitzii* group according to TLI and MH. In conclusion, these results suggest the potential of fecal microbiota as a response indicator.

## 1. Introduction

Inflammatory bowel disease (IBD) is characterized by dysregulated mucosal immune responses to the intestinal microbiota [1], although the pathogenesis is not fully understood. Tumor necrosis factor-alpha (TNF-α) monoclonal antibody, infliximab, has revolutionized IBD treatment, demonstrating its efficacy to induce clinical remission and mucosal healing (MH) in patients who were refractory to conventional therapies [2,3]. Although the direct relationship between infliximab and intestinal microbiota is not well known, it has been reported that infliximab improved gut microbial dysbiosis [4,5,6,7,8], occurring within 2–6 weeks after infliximab induction treatment [5]. Nevertheless, previous gut microbiome studies in infliximab-treated patients have shown inconsistent results, which may be due to the sensitivity of microbiota composition to biological and environmental confounders.

During maintenance therapy, infliximab is administered regularly every 8 weeks, during which serum levels steadily decrease from the peak level immediately following infliximab infusion [9,10]. Serum concentration of infliximab has been reported to have a positive association with drug efficacy in IBD patients [10]. Several studies have demonstrated its usefulness for predicting the clinical outcomes including loss of response or sustained responses, risk of colectomy, and endoscopic remission [9,11,12]. Trough level of infliximab and endoscopic mucosal healing are emerging as predictors of better clinical outcome. In practice, several patients reported symptom aggravation 1–2 weeks prior to subsequent infliximab administrations, and these symptoms recovered within 1–2 weeks after infliximab infusion. In this context, if the composition and diversity of intestinal microbiota changed meaningfully during the 8-week infliximab infusion cycle, this fluctuation of symptoms might be explained with the microbial changes according to serum infliximab level. In addition, the timing of fecal sampling after infliximab administration may be a factor to be considered when performing fecal microbial analysis.

In this study, we evaluated changes in the intestinal microbiota during an 8-week infliximab infusion cycle in IBD patients, to suggest an appropriate timing of fecal sampling for the evaluation of gut microbial composition and diversity. Furthermore, the microbial profile of patients with infliximab maintenance therapy according to emerging therapeutic targets, such as trough level of infliximab (TLI) and mucosal healing (MH), were analyzed to identify potential compositional biomarkers for IBD.

## 2. Materials and Methods

### 2.1. Study Population

This prospective longitudinal study was performed on IBD patients in clinical remission induced by infliximab (Remicade^®^, Janssen Pharmaceutical, Beerse, Belgium, or Remsima^®^, Celltrion, Incheon, Korea) and continued maintenance therapy from March 2018 to November 2018 at Samsung Medical Center in Seoul, Korea. The inclusion criteria were as follows: patients (1) were aged 18 years or more; (2) had been diagnosed with IBD (Crohn’s disease(CD) or ulcerative colitis (UC)) based on practical guidelines [13]; (3) had received intravenous administration of 5–10 mg/kg infliximab every 8 weeks at an outpatient clinic after induction at 0, 2, or 6 weeks; (4) were in clinical remission (Crohn’s disease activity index (CDAI) < 150 or partial Mayo score < 2) [14]; (5) had not concomitantly used glucocorticoids; and (6) had not used antibiotics or probiotics within 3 months. Patients who met the following criteria were excluded: (1) use of antibiotics or probiotics between the first and second fecal sampling; (2) addition or removal of IBD medication (5ASA, glucocorticoid, immunomodulator, or biologics) to an existing medication regimen; (3) altered dosage of IBD medication during study periods; (4) hospitalization or operation after enrollment; and (5) did not submit fecal samples or endoscopy. In addition, healthy adult volunteers who did not have any symptoms suggesting gastrointestinal disorder, no history of abdominal surgery, or intake of any medications including antibiotics or probiotics within 3 months were recruited as a control.

### 2.2. Study Design

This study was approved by the Institutional Review Board of Samsung Medical Center (IRB No. 2017-12-066, date of approval; 14 March 2018). All IBD patients provided written informed consent before enrollment in the study and administration with infliximab. Each patient received the first stool sampling kit, including instructions, stool collection tube containing buffer, ice pack, and insulated thermal mailers, at home using express delivery service within 1 week following infliximab infusion. Fecal samples were collected between 1 and 2 weeks after the first infliximab infusion (1W sample) and sent back to the laboratory. The second stool sampling kit was delivered to patients’ homes at 7 weeks after infliximab infusion. Patients collected their fecal samples between 7 and 8 weeks (7W sample) after infliximab infusion and sent it back to the laboratory. Fecal samples of healthy volunteers were only collected once during the study period (Ctrl sample). All fecal samples were collected with fecal collection kit, according to the manufacturer’s instructions (SMF-1; ChunLab Inc., Seoul, Korea). Samples were frozen at −80 °C until DNA extraction for 16S RNA gene sequencing and fecal calprotectin measurement. At 8 weeks after infliximab infusion, the patients’ CDAI/partial Mayo score, C-reactive protein (CRP), and TLI were assessed, followed by administration of the next infliximab treatment, as scheduled.

**Figure 1:**
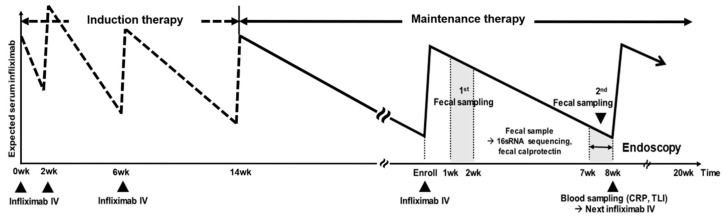
Fecal and blood sampling process. CRP: C-reactive protein, TLI: Trough level of infliximab.

All patients underwent colonoscopy (in patients with ileocolonic/colonic CD or UC) or balloon-assisted enteroscopy (in patients with ileal CD) within 3 months from the enrollment. The definition of mucosal healing (MH) was the absence of ulcerations in patients with CD and Mayo endoscopic sub-score 0 or 1 in patients with UC [15]. The sampling schedule of feces and blood is illustrated in Figure 1.

### 2.3. 16.S rRNA Gene Sequencing

Fecal microbial DNA was extracted from all submitted fecal samples using an UltraClean microbial DNA isolation kit (Mo Bio Laboratories, Carlsbad, CA, USA). The extracted DNA was stored at −80 °C until analysis. For paired-end sequencing, the hyper-variable region (V3, V4) of the bacterial 16S rRNA was amplified using 341F and 805R primers [16]. Amplicons were pooled in equal proportions and purified using the AMPure XP purification kit according to the manufacturer’s instructions. The purified product was used to prepare the Illumina DNA library. Libraries were sequenced using the Illumina MiSeq platform, generating paired-end reads of 2 × 250 bp by ChunLab Inc. (Seoul, Korea).

### 2.4. Taxonomic Comparison and Diversity Indices

Taxonomic profiling of bacterial community was analyzed using the EzBioCloud’s Microbiome Taxonomic Profiling cloud as previously described using the database version PKSSU4.0 [16]. Calculations of alpha- and beta-diversity indices, and biomarker discovery using linear discriminant analysis effect size (LEfSe) and phylogenetic investigation of communities by reconstruction of unobserved states (PICRUSt) algorithms were carried out after normalization based on 16S rRNA gene copy number variation [17,18]. Statistical testing was performed using Mann–Whitney U test. To provide community alpha-diversity estimates, species richness was assessed using Chao, ACE, Jackknife methods, and numbers of operational taxonomic units (OTUs). Diversity indices were expressed using NPShannon, Shannon, Simpson indices, and phylogenetic diversity (PD) computed from the OTU occurrence matrix. The between-sample diversity was calculated using generalized UniFrac metrics. β diversity was visualized by hierarchical cluster trees using the unweighted pair group method with arithmetic mean (UPGMA) and principal coordinate analysis (PCoA). LEfSe was employed to identify specific species that were differentially distributed between different samples, which may be available as microbial biomarkers. The linear discriminant analysis (LDA) score threshold was set to greater than 3.0 for LEfSe analysis. The functional composition of communities was described using the PICRUSt and annotated to their KEGG pathways. A *p* value of less than 0.05 was considered statistically significant.

### 2.5. Measurement of Fecal Calprotectin, CRP, and TLI

Calprotectin was measured in all submitted fecal samples using a quantitative enzyme-linked immunosorbent assay (ELISA) kit (HK379-02, Hycult Biotech, Uden, the Netherlands) according to the manufacturer’s instructions. CRP and TLI were measured at 8W following the initial infliximab and immediately before the subsequent infliximab therapy. CRP was measured by Cobas^®^ c702 (Roche Diagnostics, Mannheim, Germany), and TLI was detected using an ELISA kit (Shikari^®^ Q-Inflixi, Matriks Biotek, Ankara, Turkey). Calprotectin cut-off of > 250 μg/mL and CRP cut-off > 5 mg/L were suggested to indicate active intestinal inflammation [19]. TLI ≥ 5 µg/mL in patients with active IBD during maintenance therapy was considered as therapeutic [20].

## 3. Results

### 3.1. Study Participants and Samples

**Figure 2:**
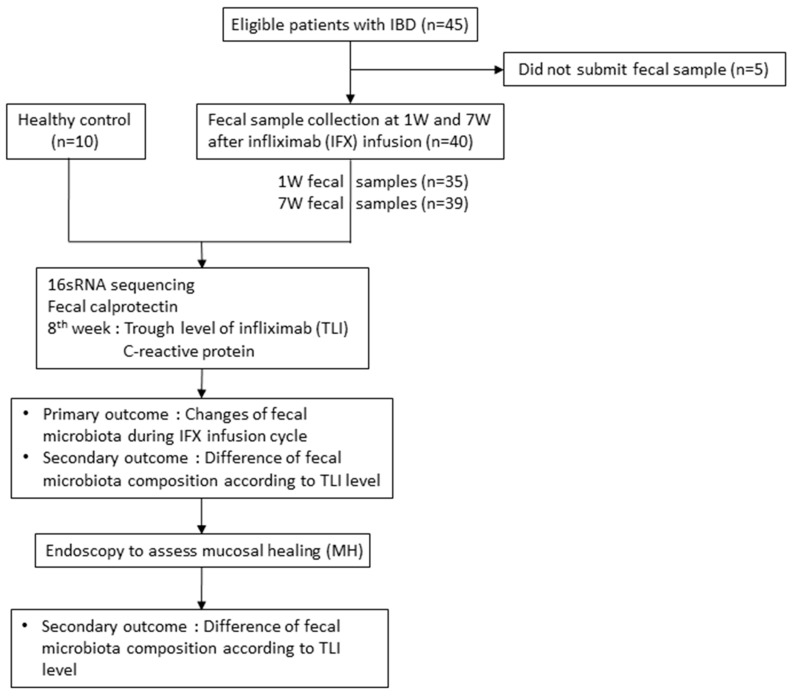
Flow diagram of the study. IBD: Inflammatory bowel disease, 1W: Fecal samples obtained at 1-2 weeks after infliximab infusion, 7W: Fecal samples obtained at 7-8 weeks after infliximab infusion.

In total, 45 IBD patients and 10 healthy volunteers were eligible for enrollment, of which 75 samples from 40 IBD patients (30 CD patients and 10 UC patients) and 10 samples from 10 healthy volunteers were analyzed (Figure 2). There was a requirement of no hospitalization, surgery, or medication changes during the study period due to symptom aggravation. A total of 34 patients submitted both 1W and 7W samples, 4 patients submitted the 1W sample only, and 2 patients submitted 7W samples only. Baseline characteristics are shown in Table 1.

Since all IBD patients were in clinical remission, calprotectin levels of all samples were < 250 μg/g, and CRP at 8 weeks after infliximab was 5 mg/L. The median level of fecal calprotectin was 4.21 μg/mL, ranging from 50 to 250 μg/g in 6 patients (15%). By contrast, the median in healthy volunteers was median 1.60 μg/g and ranged from 0.23 to 3.70 μg/g. TLI was measured in 18 patients, and 11 patients (61%) had sub-therapeutic levels of TLI. Endoscopy was performed in all enrolled patients, and MH was assessed in 12 patients with CD (50%) and 7 patients with UC (70%).

### 3.2. Changes in Microbiota Composition during 8-Week Infliximab Infusion Cycle

**Figure 3:**
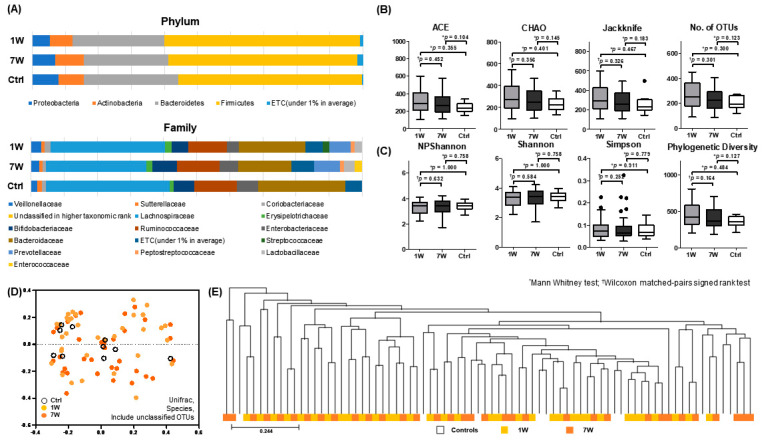
Microbial analysis of fecal samples obtained from IBD patients at 1–2 weeks (1W) and 7–8 weeks (7W) after infliximab infusion and from healthy volunteers (Ctrl)). (**A**) Stacked bar chart of microbial composition at the phylum and family level; (**B**) microbial richness assessed by ACE, CHAO, Jackknife, and number of OTUs; (**C**) biodiversity index, including NPShannon, Shannon, and Simpson indices, and phylogenetic diversity; (**D**) principal coordinate analysis (PCoA) plot; and (**E**) unweighted pair group method with arithmetic mean (UPGMA) dendrogram.

To investigate differences in microbial composition during the 8-week infusion cycle, 1W and 7W paired samples were analyzed by Wilcoxon signed-rank test; no significant differences were found in microbial composition at all ranks, including phylum and family levels (Figure 3A). Furthermore, compared to Ctrl, there were no significant differences at 1W and 7W.

Regarding average taxonomic composition at the phylum level, both 1W and 7W samples were dominated by the phyla Firmicutes and Bacteroidetes followed by Actinobacteria and Proteobacteria (Supplementary Table S1). The taxonomic relative abundance of bacterial taxa previously noted to be associated with IBD dysbiosis were compared between 1W, 7W, and Ctrl samples, which shows no meaningful differences according to timing of fecal sample collection [4,21,22].

Fecal microbial richness was not significantly different between the 1W, 7W, and Ctrl samples (Figure 3B), and the diversity indices showed no differences between samples (Figure 3C). The PCoA plot (Figure 3D) and UPGMA dendrogram (Figure 3E) were scattered randomly and showed no apparent clustering between 1W, 7W, and Ctrl samples. The LEfSe and PICRUSt analyses revealed that none of the bacterial taxa and pathways were differentially present between 1W and 7W samples.

### 3.3. Comparison of Microbiota Composition of 7W Samples with TLI < 5 μg/mL and ≥5 μg/mL

**Figure 4:**
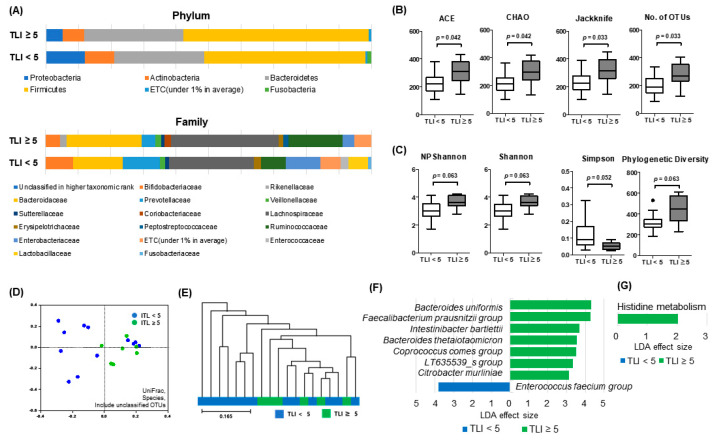
Microbial composition analysis of fecal samples obtained from IBD patients with trough level of infliximab (TLI) ≥ 5 μg/mL and TLI < 5 μg/mL. (**A**) Stacked bar chart of microbial composition at the phylum and family level; (**B**) microbial richness assessed by ACE, CHAO, Jackknife, and number of operational taxonomic units (OTUs); (**C**) biodiversity index, including NPShannon, Shannon, and Simpson indices, and phylogenetic diversity; (**D**) PCoA plot; (**E**) UPGMA dendrogram; (**F**) linear discriminant analysis effect size (LEfSe); and (**G**) phylogenetic investigation of communities by reconstruction of unobserved states (PICRUSt) analysis.

Next, we analyzed differences in microbial composition of 7W samples according to TLI. Most bacterial taxa revealed no significant differences at any ranks between TLI < 5 μg/mL and ≥ 5 μg/mL groups (Figure 4A). However, with respect to relative abundance, *Bacteroidetes uniformis*, and *Alistipes putredinis* were significantly increased and *Faecalibacterium prausnitzii* tended to increase in patients with TLI ≥ 5 μg/mL compared to TLI < 5 μg/mL (Supplementary Table S2).

Species richness represented with ACE, CHAO, and Jackknife was higher in patients with TLI ≥ 5 μg/mL than in patients with TLI < 5 μg/mL (all *p* < 0.05) (Figure 4B). Although the *p* values did not show significance, the Shannon index trended towards a significant increase in the TLI ≥ 5 μg/mL group (*p* = 0.057) and the Simpson index decreased in the TLI < 5 μg/mL group (*p* = 0.057) (Figure 4C). The PCoA plot (Figure 4D) and UPGMA dendrogram (Figure 4E) showed random scattering and no apparent clustering. The LEfSe analysis demonstrated significantly different abundances in species taxa between both groups (TLI ≥ 5 μg/mL: *Bacteroides uniformis, Faecalibacterium prausnitzii* group, *Intestinibacter bartlettii, Bacteroides thetaiotaomicron, Coprococcus comes* group, LT635539_s group, and *Citrobacter murliniae;* TLI < 5 μg/mL: *Enterococcus faecium* group), and PICRUSt analysis revealed enhanced gene allocation for histidine metabolism (Figure 4F).

### 3.4. Comparison of Microbial Profile in IBD Patients with Mucosal Healing (MH) and Patients with No Mucosal Healing (Non-MH)

**Figure 5:**
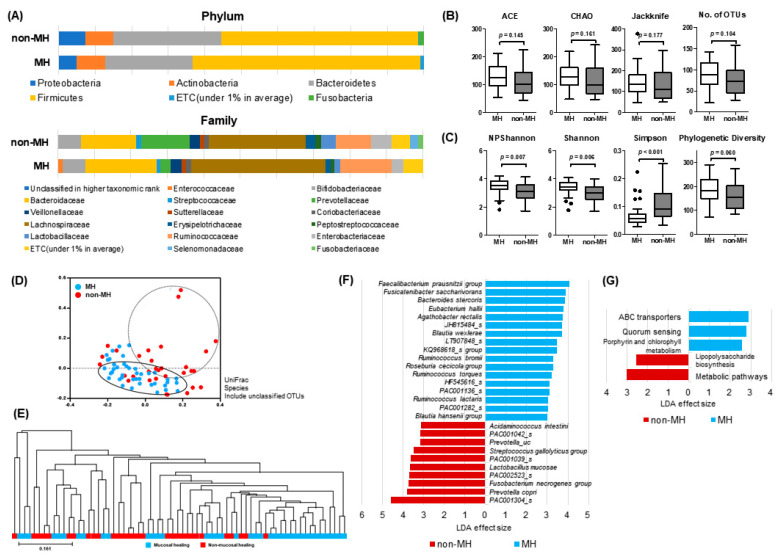
Microbial composition analysis of fecal samples obtained from IBD patients with mucosal healing (MH) and non-MH patients (**A**) Stacked bar chart of microbial composition at the phylum and family level; (**B**) microbial richness assessed by ACE, CHAO, Jackknife, and number of OTUs; (**C**) biodiversity index, including NPShannon, Shannon, and Simpson indices, and phylogenetic diversity; (**D**) PCoA plot; (**E**) UPGMA dendrogram; (**F**) LEfSe; and (**G**) PICRUSt analysis.

Microbial profiles in IBD patients were analyzed according to MH. The average taxonomic composition of phylum Firmicutes and Bacteroidetes of the MH group was increased and decreased compared with the non-MH group, respectively (Figure 5A). In addition, relative abundance of some IBD-associated bacterial taxa showed significant differences between MH and non-MH groups (Supplementary Table S3). In particular, the MH group showed significantly higher abundance of genera *Faecalibacterium, Blautia*, and *Bacteroides*, and lower abundance of *Prevotella*. Furthermore, the B/P (*Bacteroides* to *Prevotella*) ratio of the MH group was significantly higher than that of non-MH group (*p* = 0.001), and closer to the healthy control group (Supplementary Figure S1).

Biodiversity indices, including NPShannon, Shannon, Simpson, and PD, showed significant differences between both groups, indicating higher species richness and evenness in patients with MH (Figure 5C). Species richness represented by ACE, Chao and Jackknife showed an increased tendency in the MH group (Figure 5B).

On the PCoA plot and UPGMA dendrogram (Figure 5D,E), MH and non-MH groups tended to distribute separately. LEfSe analysis demonstrated significantly-different abundances of the following species taxa in both groups: MH—*Faecalibacterium prausnitzii* group, *Fusicatenibacter saccharivorans, Bacteroides stercoris, Eubacterium hallii, Agathobacter rectalis*, JH815484, *Blautia wexlerae*, LT907848_s, KQ968618_s group, *Ruminococcus bromii, Roseburia cecicola* group, *Ruminococcus torques*, HF545616_s. PAC001136_s, *Ruminococcus lactaris*, PAC001282_s, and *Blautia hansenii* group; non-MH group—PAC001304_s, *Prevotella copri, Fusobacterium necrogenes* group, PAC002523_s, *Lactobacillus mucosae*, PAC001039_s, *Streptococcus gallolyticus* group, *Prevotella_uc*, PAC001042, and *Acidaminococcus intestine* (Figure 5F). PICRUSt analysis revealed enhanced gene allocation for ABC transporters, quorum sensing, and porphyrin and chlorophyll metabolism in the MH group, whereas lipopolysaccharide (LPS) biosynthesis were increased in the non-MH group (Figure 5G).

## 4. Discussion

In this prospective study, infliximab showed little effect on fecal microbiota composition in IBD patients with clinical remission during an 8-week infusion cycle. Further, there were no significant differences between 1W and 7W samples in terms of biodiversity. However, our study found that fecal microbiota in patients with therapeutic TLI and MH showed increased bacterial diversity, richness, and relative abundance of *F. prausnitzii* group, compared with the patients with subtherapeutic TLI and non-MH. These results suggest that the composition and biodiversity of gut microbiota is related to the treatment response of anti-TNF agents, as represented by therapeutic TLI and MH.

It is well established that bacterial diversity and richness of the intestinal microbiota are reduced in active IBD patients [7,23], whereas dysbiosis is diminished in inactive IBD patients after infliximab treatment [4,5,6,7,8]. However, as serum infliximab concentration is decreased steadily after infusion [10], drug efficacy decreases, and some patients without non-MH or deep remission experience fluctuations of their symptoms according to the infliximab infusion cycle. Previous gut microbiome studies have not evaluated microbiota changes during an 8-week infliximab treatment. Therefore, we assumed that microbiota would change over time after infliximab infusion as serum infliximab concentrations changed; however, there were no distinguishable differences in composition and diversity between 1W and 7W fecal samples. Furthermore, there was no significant difference between the Ctrl and 1W or 7W samples, which is similar to a previous report [7].

Temporal instability can occur even in healthy people. Therefore, more specific and distinct differences would be needed to differentiate changes between 1W and 7W that would be greater than normal variation. In practice, this result indicated that the timing of fecal sampling is not essential in patients with clinical remission when evaluating microbial composition and diversity during infliximab maintenance therapy. However, dysbiosis in microbial richness was significantly decreased, and some taxa were significantly changed in the TLI < 5 μg/g group. LefSe analysis identified several taxonomic markers, such as *Bacteroides uniformis* and *F. prausnitzii*, for therapeutic TLI.

Treat-to-target strategy to achieve mucosal healing is a current concept used for IBD. Regardless of sampling points, the intestinal microbiota between MH and non-MH samples showed greater heterogeneity in microbial community diversity. Fecal samples assigned to the non-MH group showed decreased microbial biodiversity and differences in relative abundance of multiple taxa compared to MH samples. In previous studies, the proportion of Firmicutes phylum, especially *F. prausnitzii*, was reduced, and Bacteroidetes, including *Enterobacteriaceae* and *E. coli*, was increased in CD patients with high disease activity. Further, dysbiosis of intestinal microbiota is observed in highly-active CD patients compared to healthy controls and remission patients [24,25]. Similarly, our study showed that phyla Firmicutes and Bacteroidetes were significantly decreased and increased in non-MH group, respectively, compared to MH group. At the species level, relative abundance of *F. prausnitzii* and *Prevotella* PAC001304_s were significantly different.

There is great interest in developing the gut microbiome as a clinical diagnostic test for disease severity and for monitoring treatment response for IBD, although previous gut microbiome studies could not derive taxonomic biomarkers [26]. In this study, to reduce the impact of major confounding factors, we enrolled adult IBD patients with clinical remission as well as normal CRP and fecal calprotectin levels, because disease activity is a major influential factor for dysbiosis [27]. LefSe analysis identified *F. prausnitzii* as a microbial biomarker for therapeutic TLI and MH. *F. prausnitzii* is one of the most important butyrate-producing bacteria and plays a role in prebiotic fermentation [28]. It represents around 5% of total bacteria in stool samples from healthy adults, and in vitro peripheral blood mononuclear cell stimulation by *F. prausnitzii* leads to significantly lower IL-12 and IFN-γ production and higher IL-10 secretion [29,30]. Further, increased *F. prausnitzii* has been demonstrated in patients treated with infliximab [31]. In PICRUSt analysis, histidine metabolism was associated with 7W fecal samples of therapeutic TLI. Histidine produces butyrate through colonic microbiota [32], inhibiting oxidative stress- and TNF-α-induced IL-8 secretion in intestinal epithelial cells [33]. Conversely lipopolysaccharide biosynthesis were increased in non-MH samples. Lipopolysaccharide is the major component of the outer membrane of Gram-negative bacteria and acts as the prototypical endotoxin in inflammatory cells, which promotes the secretion of pro-inflammatory cytokines, nitric oxide, and eicosanoids [34]. Increased LPS biosynthesis could suggest overgrowth of gram negative bacteria. These bacterial species and metabolic targets have potential to be used as future biomarkers of MH or therapeutic boosters that promote MH with anti-TNF agents.

However, our study had some limitations. First, the study population was relatively small and from a single center. To exclude disease activity which is the major confounding factor for gut microbiome, we enrolled only patients in clinical remission. However, although both are classified as IBD, microbial profiles of CD and UC are different from each other. In particular, the number of UC patients was only 10, therefore, further analysis is required using a large number of patients and an accordant sample size to further validate that there are no differences between the groups. Second, although we analyzed bacteria only based on 16S rRNA sequencing, gut microbiota encompass bacteria, viruses, phages, and fungi [35]. In addition, as sequencing technologies can only provide gut composition, we also need to understand the functional effects of microbiota on the complex networks of the intestinal immune system. Although mucosa-adherent bacteria reflect an imbalance of microbial community more directly than fecal bacteria [27,36], luminal samples are more feasible and practical biomarkers due to their convenience and noninvasiveness.

In conclusion, in patients with clinical remission during infliximab maintenance therapy, no significant differences in microbial composition and diversity were observed, and the timing of fecal sampling did not appear to be important when evaluating the intestinal microbiota. However, microbial composition and diversity differed according to the TLI and MH status. Therefore, changes in the intestinal microbiota composition and biodiversity might be used to predict therapeutic TLI and MH in IBD patients treated with infliximab. To confirm utility as a potential response indicator, it should be compared against other markers, such as infliximab trough levels or fecal calprotectin.

## Figures and Tables

**Table 1:** Baseline characteristics of enrolled IBD patients.

	Crohn’s Disease (*n* = 30)	Ulcerative Colitis (*n* = 10)	p *	IBD (*n* = 40)	Healthy Control (*n* = 10)	*p* ^†^
Age (years), median (IQR)	33.0 (25.7–42.2)	48.0 (36.0–51.5)	0.016	37.5 (27.8–48.0)	30.5 (27.00–33.5)	0.074
Male, *n* (%)	25 (83.3)	4 (40.0)	0.008	29 (72.5)	6 (60.0)	0.440
Current smoker, *n* (%)	1 (4.2)	0 (0)	1.000	1 (2.9)	0 (0)	1.000
Abdominal surgery, *n* (%)	7 (29.2)	0 (0–0)	0.078	7 (20.6)	0 (0–0)	0.161
Montreal classification						
A1/A2/A3, *n* (%)	2 (8.3)/22 (91.7)/0 (0)	-				
L1/L2/L3, *n* (%)	4 (16.7)/5 (20.8)/15 (62.5)	-				
B1/B2/B3, *n* (%)	10 (41.7)/11 (45.8)/3 (12.5)	-				
E1/E2/E3, *n* (%)	-	2 (20.0)/2 (20.0)/6 (60.0)				
Azathiopurine use, *n* (%)	11 (45.8)	1 (10.0)		12 (35.3)		
BMI (kg/m^2^), median (IQR)	21.6 (19.0–24.0)	22.4 (20.5–24.7)	0.322	21.8 (19.7–24.4)		
Hemoglobin (g/dL), median (IQR)	13.0 (11.6–15.0)	13.7 (12.9–14.9)	0.589	13.3 (11.9–14.9)		
Hematocrit (%), median (IQR)	39.8 (37.2–44.4)	42.3 (40.3–44.7)	0.539	42.1 (37.6–44.6)		
Albumin (g/dL), median (IQR)	4.6 (4.3–4.7)	4.6 (4.4–4.70)	0.411	4.6 (4.3–4.7)		
ESR (mm/hr), median (IQR)	8.5 (5.0–19.5)	14.0 (6.5–41.0)	0.752	10.5 (5.3–20.5)		
CRP (mg/L), median (IQR)	0.5 (0.3–2.6)	0.6 (0.3–1.2)	0.752	0.6 (0.3–1.6)		
Calprotectin (μg/mL), median (IQR)	19.8 (3.0–41.0)	2.2 (1.7–51.8)	0.006	4.21 (1.9–38.2)	1.60 (0.78–2.72)	0.001
Trough level of Infliximab (μg/mL), median (IQR)	4.3 (3.2–5.8)	4.8 (4.3–6.4)	0.534	4.4 (3.4–5.9)		
Mucosal healing (*n*, %)	12 (50.0)	7 (70.0)	0.451	19 (55.9)		

IBD: inflammatory bowel disease, ESR: erythrocyte sedimentation rate, CRP: C-reactive protein, IQR: interquartile range. * Crohn’s disease vs. ulcerative colitis; ^†^ IBD vs. healthy control.

## Supplementary Materials

The following are available online at https://www.mdpi.com/2076-2607/8/6/874/s1, Table S1. Taxonomic relative abundance of bacterial taxa associated with IBD; Table S2. Taxonomic relative abundance of bacterial taxa associated with IBD according to trough level of infliximab (TLI); Table S3. Comparison of taxonomic relative abundance of bacterial taxa associated with IBD between mucosal healing (MH) and non-mucosal healing (non-MH) groups; Figure S1. *Bacteroides* to *Prevotella* ratio in healthy control, patients with mucosal healing (MH) and non-mucosal healing (non-MH) groups.

## References

[1] Wallace K.L., Zheng L.B., Kanazawa Y., Shih D.Q. (2014). Immunopathology of inflammatory bowel disease. World J. Gastroenterol..

[2] Busquets D., Mas-de-Xaxars T., Lopez-Siles M., Martinez-Medina M., Bahi A., Sabat M., Louvriex R., Miquel-Cusachs J.O., Garcia-Gil J.L., Aldeguer X. (2015). Anti-tumour Necrosis Factor Treatment with Adalimumab Induces Changes in the Microbiota of Crohn’s Disease. J. Crohns Colitis.

[3] Bazin T., Hooks K.B., Barnetche T., Truchetet M.E., Enaud R., Richez C., Dougados M., Hubert C., Barre A., Nikolski M. (2018). Microbiota Composition May Predict Anti-Tnf Alpha Response in Spondyloarthritis Patients: An Exploratory Study. Sci. Rep..

[4] Rajca S., Grondin V., Louis E., Vernier-Massouille G., Grimaud J.C., Bouhnik Y., Laharie D., Dupas J.L., Pillant H., Picon L. (2014). Alterations in the intestinal microbiome (dysbiosis) as a predictor of relapse after infliximab withdrawal in Crohn’s disease. Inflamm. Bowel Dis..

[5] Magnusson M.K., Strid H., Sapnara M., Lasson A., Bajor A., Ung K.A., Ohman L. (2016). Anti-TNF Therapy Response in Patients with Ulcerative Colitis Is Associated with Colonic Antimicrobial Peptide Expression and Microbiota Composition. J. Crohns Colitis.

[6] Eun C.S., Kwak M.J., Han D.S., Lee A.R., Park D.I., Yang S.K., Kim Y.S., Kim J.F. (2016). Does the intestinal microbial community of Korean Crohn’s disease patients differ from that of western patients?. BMC Gastroenterol..

[7] Wang Y., Gao X., Ghozlane A., Hu H., Li X., Xiao Y., Li D., Yu G., Zhang T. (2018). Characteristics of Faecal Microbiota in Paediatric Crohn’s Disease and Their Dynamic Changes During Infliximab Therapy. J. Crohns Colitis.

[8] Zhou Y., Xu Z.Z., He Y., Yang Y., Liu L., Lin Q., Nie Y., Li M., Zhi F., Liu S. (2018). Gut Microbiota Offers Universal Biomarkers across Ethnicity in Inflammatory Bowel Disease Diagnosis and Infliximab Response Prediction. mSystems.

[9] Koga A., Matsui T., Takatsu N., Takada Y., Kishi M., Yano Y., Beppu T., Ono Y., Ninomiya K., Hirai F. (2018). Trough level of infliximab is useful for assessing mucosal healing in Crohn’s disease: A prospective cohort study. Intest. Res..

[10] Adedokun O.J., Sandborn W.J., Feagan B.G., Rutgeerts P., Xu Z., Marano C.W., Johanns J., Zhou H., Davis H.M., Cornillie F. (2014). Association between serum concentration of infliximab and efficacy in adult patients with ulcerative colitis. Gastroenterology.

[11] Bortlik M., Duricova D., Malickova K., Machkova N., Bouzkova E., Hrdlicka L., Komarek A., Lukas M. (2013). Infliximab trough levels may predict sustained response to infliximab in patients with Crohn’s disease. J. Crohns Colitis.

[12] Seow C.H., Newman A., Irwin S.P., Steinhart A.H., Silverberg M.S., Greenberg G.R. (2010). Trough serum infliximab: A predictive factor of clinical outcome for infliximab treatment in acute ulcerative colitis. Gut.

[13] Ye B.D., Jang B.I., Jeen Y.T., Lee K.M., Kim J.S., Yang S.K. (2009). Diagnostic guideline of Crohn’s disease. Korean J. Gastroenterol..

[14] Choi C.H., Kim Y.H., Kim Y.S., Ye B.D., Lee K.M., Lee B.I., Jung S.A., Kim W.H., Lee H. (2012). Guidelines for the management of ulcerative colitis. Korean J. Gastroenterol..

[15] Levin A.D., Wildenberg M.E., van den Brink G.R. (2016). Mechanism of Action of Anti-TNF Therapy in Inflammatory Bowel Disease. J. Crohns Colitis.

[16] Yoon S.H., Ha S.M., Kwon S., Lim J., Kim Y., Seo H., Chun J. (2017). Introducing EzBioCloud: A taxonomically united database of 16S rRNA gene sequences and whole-genome assemblies. Int. J. Syst. Evol. Microbiol..

[17] Segata N., Izard J., Waldron L., Gevers D., Miropolsky L., Garrett W.S., Huttenhower C. (2011). Metagenomic biomarker discovery and explanation. Genome Biol..

[18] Langille M.G., Zaneveld J., Caporaso J.G., McDonald D., Knights D., Reyes J.A., Clemente J.C., Burkepile D.E., Vega Thurber R.L., Knight R. (2013). Predictive functional profiling of microbial communities using 16S rRNA marker gene sequences. Nat. Biotechnol..

[19] Colombel J.F., Panaccione R., Bossuyt P., Lukas M., Baert F., Vanasek T., Danalioglu A., Novacek G., Armuzzi A., Hebuterne X. (2018). Effect of tight control management on Crohn’s disease (CALM): A multicentre, randomised, controlled phase 3 trial. Lancet.

[20] Feuerstein J.D., Nguyen G.C., Kupfer S.S., Falck-Ytter Y., Singh S., American Gastroenterological Association Institute Clinical Guidelines Committee (2017). American Gastroenterological Association Institute Guideline on Therapeutic Drug Monitoring in Inflammatory Bowel Disease. Gastroenterology.

[21] Pascal V., Pozuelo M., Borruel N., Casellas F., Campos D., Santiago A., Martinez X., Varela E., Sarrabayrouse G., Machiels K. (2017). A microbial signature for Crohn’s disease. Gut.

[22] Zuo T., Ng S.C. (2018). The Gut Microbiota in the Pathogenesis and Therapeutics of Inflammatory Bowel Disease. Front. Microbiol..

[23] Qin J., Li R., Raes J., Arumugam M., Burgdorf K.S., Manichanh C., Nielsen T., Pons N., Levenez F., Yamada T. (2010). A human gut microbial gene catalogue established by metagenomic sequencing. Nature.

[24] Tedjo D.I., Smolinska A., Savelkoul P.H., Masclee A.A., van Schooten F.J., Pierik M.J., Penders J., Jonkers D.M. (2016). The fecal microbiota as a biomarker for disease activity in Crohn’s disease. Sci. Rep..

[25] Walker A.W., Sanderson J.D., Churcher C., Parkes G.C., Hudspith B.N., Rayment N., Brostoff J., Parkhill J., Dougan G., Petrovska L. (2011). High-throughput clone library analysis of the mucosa-associated microbiota reveals dysbiosis and differences between inflamed and non-inflamed regions of the intestine in inflammatory bowel disease. BMC Microbiol..

[26] Knox N.C., Forbes J.D., Van Domselaar G., Bernstein C.N. (2019). The Gut Microbiome as a Target for IBD Treatment: Are We There Yet?. Curr. Treat. Options Gastroenterol..

[27] Gevers D., Kugathasan S., Denson L.A., Vazquez-Baeza Y., Van Treuren W., Ren B., Schwager E., Knights D., Song S.J., Yassour M. (2014). The treatment-naive microbiome in new-onset Crohn’s disease. Cell Host Microbe.

[28] Ferreira-Halder C.V., Faria A.V.S., Andrade S.S. (2017). Action and function of Faecalibacterium prausnitzii in health and disease. Best Pract. Res. Clin. Gastroenterol..

[29] Louis P., Flint H.J. (2009). Diversity, metabolism and microbial ecology of butyrate-producing bacteria from the human large intestine. FEMS Microbiol. Lett..

[30] Sokol H., Pigneur B., Watterlot L., Lakhdari O., Bermudez-Humaran L.G., Gratadoux J.J., Blugeon S., Bridonneau C., Furet J.P., Corthier G. (2008). Faecalibacterium prausnitzii is an anti-inflammatory commensal bacterium identified by gut microbiota analysis of Crohn disease patients. Proc. Natl. Acad. Sci. USA.

[31] Schaffler H., Kaschitzki A., Alberts C., Bodammer P., Bannert K., Koller T., Warnke P., Kreikemeyer B., Lamprecht G. (2016). Alterations in the mucosa-associated bacterial composition in Crohn’s disease: A pilot study. Int. J. Colorectal Dis..

[32] Rowland I., Gibson G., Heinken A., Scott K., Swann J., Thiele I., Tuohy K. (2018). Gut microbiota functions: Metabolism of nutrients and other food components. Eur. J. Nutr..

[33] Son D.O., Satsu H., Shimizu M. (2005). Histidine inhibits oxidative stress- and TNF-alpha-induced interleukin-8 secretion in intestinal epithelial cells. FEBS Lett..

[34] Guerville M., Boudry G. (2016). Gastrointestinal and hepatic mechanisms limiting entry and dissemination of lipopolysaccharide into the systemic circulation. Am. J. Physiol. Gastrointest. Liver Physiol..

[35] Neish A.S. (2009). Microbes in gastrointestinal health and disease. Gastroenterology.

[36] Burrough E.R., Arruda B.L., Plummer P.J. (2017). Comparison of the Luminal and Mucosa-Associated Microbiota in the Colon of Pigs with and without Swine Dysentery. Front. Vet. Sci..

