# Supplementary material for: Changes in the Intestinal Microbiota of Patients with Inflammatory Bowel Disease with Clinical Remission during an 8-Week Infliximab Infusion Cycle
Source: Microorganisms. 2020 Jun 9;8(6):874. doi: 10.3390/microorganisms8060874 (PMC7356282; doi:10.3390/microorganisms8060874)
Supplement: Supplementary file 1 [file microorganisms-08-00874-s001.zip › Supple_Table_micro.docx]

**Supplemental Table 1.** Taxonomic relative abundance of bacterial taxa associated with IBD.

|  | **1W (n = 34)** | **7W (n = 34)** | **p*** | **Ctrl** | **p†** | **p§** |
| --- | --- | --- | --- | --- | --- | --- |
| [P] Firmicutes, %, median (IQR) | 57.22 (49.98-69.75) | 59.06 (46.51-67.23) | 0.668 | 57.54 (49.13-67.05) | 0.634 | 0.779 |
| [G] *Faecalibacterium* (%) | 4.93 (0.06-10.28) | 4.69 (0.07-10.24) | 0.650 | 6.69 (1.88-10.12) | 0.313 | 0.370 |
| [S] *Faecalibacterium prausnitzii group*, %, median (IQR) | 4.80 (0.06-10.28) | 4.69 (0.06-9.13) | 0.704 | 6.68 (1.88-10.02) | 0.300 | 0.287 |
| [G] *Oscillibacter*, %, median (IQR) | 0.30 (0.01-1.39) | 0.56 (0.03-1.19) | 0.677 | 0.19 (0.03-1.18) | 0.955 | 0.716 |
| [G] *Agathobacter*, %, median (IQR) | 0.20 (0.00-1.64) | 0.20 (0.00-1.39) | 0.768 | 0.04 (0.00-0.26) | 0.385 | 0.287 |
| [G] *Blautia*, %, median (IQR) | 8.14 (4.77-12.08) | 7.39 (4.49-9.63) | 0.275 | 9.46 (6.83-13.83) | 0.341 | 0.073 |
| [S] *Blautia wexlerae*, %, median (IQR) | 2.95 (1.18-8.09) | 3.35 (0.93-4.12) | 0.686 | 4.07 (2.74-6.00) | 0.484 | 0.370 |
| [G] *Clostridium*, %, median (IQR) | 0.18 (0.02-1.22) | 0.18 (0.01-0.68) | 0.990 | 0.03 (0.01-0.18) | 0.157 | 0.170 |
| [S] *Clostridium perfringens*, %, median (IQR) | 0.00 (0.00-0.02) | 0.00 (0.00-0.01) | 0.425 | 0.00 (0.00-0.00) | 0.218 | 0.450 |
| [P] Bacteroidetes, %, median (IQR) | 24.78 (16.08-43.64) | 24.80 (17.08-35.58) | 0.713 | 25.46 (16.26-39.63) | 1.000 | 0.779 |
| [G] *Alistipes*, %, median (IQR) | 0.04 (0.00-0.03) | 0.12 (0.00-1.00) | 0.768 | 0.42 (0.00-0.73) | 0.654 | 0.922 |
| [S] *Alistipes putredinis*, %, median (IQR) | 0.00 (0.00-0.30) | 0.00 (0.00-0.53) | 0.484 | 0.00 (0.00-0.27) | 0.801 | 0.519 |
| [G] *Barnesiella*, %, median (IQR) | 0.00 (0.00-0.00) | 0.00 (0.00-0.00) | 0.759 | 0.04 (0.00-0.23) | 0.069 | 0.069 |
| [G] *Bacteroides*, %, median (IQR) | 18.26 (4.85-33.31) | 14.66 (2.80-23.62) | 0.320 | 23.71 (14.01-36.20) | 0.287 | 0.093 |
| [S] *Bacteroides uniformis*, %, median (IQR) | 0.27 (0.01-1.61) | 0.46 (0.01-1.91) | 0.859 | 0.88 (0.43-1.92) | 0.153 | 0.161 |
| [G] *Prevotella*, %, median (IQR) | 0.03 (0.01-0.47) | 0.04 (0.02-2.8) | 0.820 | 0.02 (0.01-0.03) | 0.287 | 0.157 |
| [S] *PAC001304*, %, median (IQR) | 0.01 (0.00-0.46) | 0.01 (0.00-0.66) | 0.859 | 0.01 (0.00-0.01) | 0.240 | 0.145 |
| [P] Actinobacteria, %, median (IQR) | 5.40 (1.30-9.70) | 5.95 (1.50-11.7) | 0.792 | 8.58 (2.00-12.23) | 0.614 | 0.955 |
| [G] *Bifidobacterium*, %, median (IQR) | 2.67 (1.01-7.04) | 4.36 (0.69-10.42) | 0.556 | 7.35 (1.34-10.71) | 0.417 | 0.867 |
| [S] *Bifidobacterium adolescentis group*, %, median (IQR) | 0.12 (0.00-3.16) | 0.27 (0.00-3.57) | 0.759 | 0.01 (0.00-9.39) | 0.695 | 0.566 |
| [P] Proteobacteria, %, median (IQR) | 3.86(2.27-7.04) | 3.13(1.87-9.74) | 0.922 | 4.96(2.69-9.03) | 0.556 | 0.370 |
| [G] *Escherichia*, %, median (IQR) | 0.69 (0.04-2.27) | 0.42 (0.05-2.56) | 0.820 | 0.50 (0.07-2.07) | 0.737 | 0.634 |
| [S] *Escherichia coli group*, %, median (IQR) | 0.69 (0.04-2.27) | 0.42 (0.05-2.56) | 0.820 | 0.50 (0.07-2.07) | 0.737 | 0.634 |

1W: fecal samples obtained from IBD patients between 1 and 2 weeks after IFX infusion, 7W: fecal samples obtained from IBD patients between 7 and 8 weeks after IFX infusion, Ctrl: fecal sample obtained from healthy volunteers, IQR: interquartile range, P: Phylum, G: Genus, S: Species.

*Wilcoxon signed-rank test between 1W and 7W;

†Mann-Whitney U test between 1W and Ctrl;

§Mann-Whitney U test between 7W and Ctrl.

**Supplemental Table 2.** Taxonomic relative abundance of bacterial taxa associated with IBD according to trough level of infliximab (TLI).

|  | **TLI ≥ 5 µg/mL (n = 7)** | **TLI < 5 µg/mL (n = 11)** | **p*** |
| --- | --- | --- | --- |
| [P] Firmicutes, %, median (IQR) | 57.91 (52.15-61.39) | 49.69 (36.07-61.14) | 0.258 |
| [G] *Faecalibacterium* (%) | 7 87 (2.17-9.94) | 0.06 (0.02-4.8) | 0.052 |
| [S] *Faecalibacterium prausnitzii group*, %, median (IQR) | 7.64 (2.17-9.94) | 0.06 (0.02-4.8) | 0.052 |
| [G] *Oscillibacter*, %, median (IQR) | 0.67 (0.41-1.92) | 0.09 (0.01-0.8) | 0.160 |
| [G] *Agathobacter*, %, median (IQR) | 0.37 (0.05-1.05) | 0.01 (0.00-1.8) | 1.000 |
| [G] *Blautia*, %, median (IQR) | 8.02 (7.28-10.22) | 5.86 (2.76-9.61) | 0.342 |
| [S] *Blautia wexlerae*, %, median (IQR) | 4.60 (2.23-6.94) | 2.96 (0.99-5.67) | 0.298 |
| [G] *Clostridium*, %, median (IQR) | 0.38 (0.19-0.86) | 0.16 (0.02-0.67) | 0.526 |
| [S] *Clostridium perfringens*, %, median (IQR) | 0.00 (0.00-0.07) | 0.00 (0.00-0.04) | 0.651 |
| [P] Bacteroidetes, %, median (IQR) | 28.45 (22.29-34.47) | 30.06(13.95-44.68) | 0.684 |
| [G] *Alistipes*, %, median (IQR) | 0.41 (0.05-3.47) | 0.00 (0.00-0.52) | 0.077 |
| [S] *Alistipes putredinis*, %, median (IQR) | 0.29 (0.00-2.5) | 0.00 (0.00-0.00) | 0.037 |
| [G] *Barnesiella*, %, median (IQR) | 0.00 (0.00-0.00) | 0.00 (0.00-0.00) | 0.821 |
| [G] *Bacteroides*, %, median (IQR) | 21.34 (16.26-30.35) | 8.04 (1.28-23.19) | 0.160 |
| [S] *Bacteroides uniformis*, %, median (IQR) | 4.19 (0.94-7.59) | 0.02 (0.01-1.42) | 0.026 |
| [G] *Prevotella*, %, median (IQR) | 0.02 (0.01-0.06) | 0.04 (0.02-16.93) | 0.221 |
| [S] *PAC001304*, %, median (IQR) | 0.00 (0.00-0.02) | 0.03 (0.01-11.72) | 0.258 |
| [G] *Bacteroides/Prevotella* ratio, Log(%), median (IQR) | 2.82(2.41-3.56) | 3.04(-1.00-3.39) | 0.724 |
| [P] Actinobacteria, %, median (IQR) | 4.46 (1.81-9.32) | 4.63.00 (0.64-6.06) | 0.556 |
| [G] *Bifidobacterium*, %, median (IQR) | 2.54 (1.12-4.42) | 3.29 (0.40-5.64) | 0.964 |
| [S] *Bifidobacterium adolescentis group*, %, median (IQR) | 0.29 (0.00-1.92) | 0.03 (0.00-2.67) | 0.928 |
| [P] Proteobacteria, %, median (IQR) | 2.26 (1.93-6.24) | 9.93(5.93-10.76) | 0.077 |
| [G] *Escherichia*, %, median (IQR) | 1.65 (0.24-1.88) | 2.62 (0.25-5.10) | 0.221 |
| [S] *Escherichia coli group*, %, median (IQR) | 1.65 (0.24-1.88) | 2.62 (0.25-5.10) | 0.221 |

TLI: trough level of infliximab, IQR: interquartile range.

*****Mann-Whitney U test

**Supplemental Table 3.** Comparison of taxonomic relative abundance of bacterial taxa associated with IBD between mucosal healing (MH) and non-mucosal healing (non-MH) groups.

|  | **MH (n = 42)** | **non-MH (n = 32)** | **p*** |
| --- | --- | --- | --- |
| [P] Firmicutes, %, median (IQR) | 63.40 (55.10-71.00) | 55.55 (43.35-63.00) | 0.117 |
| [G] *Faecalibacterium* (%) | 8.70 (0.80-10.90) | 1.00 (0.10-7.05) | 0.018 |
| [S] *Faecalibacterium prausnitzii group*, %, median (IQR) | 8.40 (0.80-10.90) | 1.00 (0.10-6.50) | 0.013 |
| [G] *Oscillibacter*, %, median (IQR) | 0.50 (0.10-1.40) | 0.10 (0.00-1.05) | 0.174 |
| [G] *Agathobacter*, %, median (IQR) | 0.80 (0.00-2.70) | 0.05 (0.00-0.30) | 0.014 |
| [G] *Blautia*, %, median (IQR) | 7.90 (6.00-10.80) | 5.50 (1.50-9.65) | 0.045 |
| [S] *Blautia wexlerae*, %, median (IQR) | 3.65 (2.40-6.00) | 1.45 (0.50-4.20) | 0.032 |
| [G] *Clostridium*, %, median (IQR) | 0.10 (0.00-0.80) | 0.20 (0.00-0.80) | 0.909 |
| [S] *Clostridium perfringens*, %, median (IQR) | 0.00 (0.00-0.00) | 0.00 (0.00-0.00) | 0.326 |
| [P] Bacteroidetes, %, median (IQR) | 23.0 (15.30-33.40) | 30.2 (19.7-44.35) | 0.153 |
| [G] *Alistipes*, %, median (IQR) | 0.15 (0.00-1.3) | 0.05 (0.00-0.55) | 0.631 |
| [S] *Alistipes putredinis*, %, median (IQR) | 0.00 (0.00-0.50) | 0.00 (0.00-0.40) | 0.956 |
| [G] *Barnesiella*, %, median (IQR) | 0.00 (0.00-0.00) | 0.00 (0.00-0.00) | 0.541 |
| [G] *Bacteroides*, %, median (IQR) | 16.85 (7.50-27.40) | 6.30 (1.65-24.60) | 0.047 |
| [S] *Bacteroides uniformis*, %, median (IQR) | 0.20 (0.00-1.90) | 0.25 (0.00-1.95) | 0.900 |
| [G] *Prevotella*, %, median (IQR) | 0.00 (0.00-0.10) | 0.35 (0.00-28.25) | 0.004 |
| [S] *PAC001304*, %, median (IQR) | 0.0 (0.0-0.0) | 0.25 (0.00-19.85) | 0.002 |
| [G] *Bacteroides/Prevotella* ratio, Log(%), median (IQR) | 2.98(2.1-3.41) | 0.76(-1.04-2.91) | 0.001 |
| [P] Actinobacteria, %, median (IQR) | 6.45 (2.50-11.30) | 3.90 (1.15-8.20) | 0.198 |
| [G] *Bifidobacterium*, %, median (IQR) | 4.90 (2.20-8.70) | 2.55 (0.40-6.40) | 0.081 |
| [S] *Bifidobacterium adolescentis group*, %, median (IQR) | 0.05 (0.00-4.10) | 0.20 (0.00-2.10) | 0.853 |
| [P] Proteobacteria, %, median (IQR) | 2.80 (1.90-5.90) | 4.75 (2.30-8.85) | 0.110 |
| [G] *Escherichia*, %, median (IQR) | 0.55 (0.10-1.80) | 1.25 (0.00-4.60) | 0.627 |
| [S] *Escherichia coli group*, %, median (IQR) | 0.55 (0.10-1.80) | 1.25 (0.00-4.60) | 0.620 |

MH: mucosal healing, non-MH: non-mucosal healing, IQR: interquartile range.

*****Mann-Whitney U test
