# Supplementary figures and images for: Changes in the Intestinal Microbiota of Patients with Inflammatory Bowel Disease with Clinical Remission during an 8-Week Infliximab Infusion Cycle
Source: Microorganisms. 2020 Jun 9;8(6):874. doi: 10.3390/microorganisms8060874 (PMC7356282; doi:10.3390/microorganisms8060874)

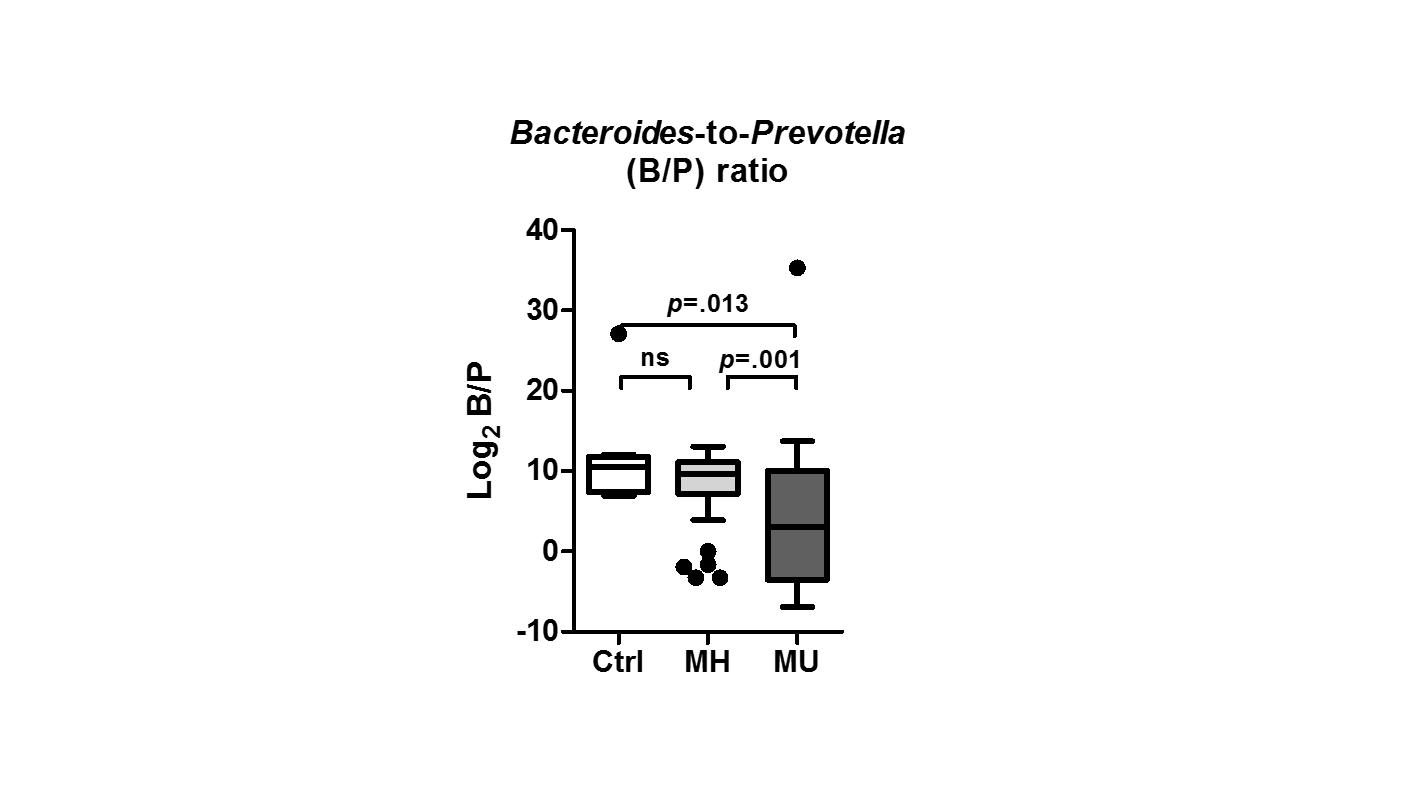

Supplement: Supplementary file 1 [file microorganisms-08-00874-s001.zip › Supplementary Figure 1_micro.tif]
